# Detection of Antibodies to *Chlamydia trachomatis* With Peptide-Based Species-Specific Enzyme Immunoassay

**DOI:** 10.1155/S1064744997000616

**Published:** 1997

**Authors:** Ale Närvänen, Mirja Puolakkainen, Wu Hao, Kohsuke Kino, Jukka Suni

**Affiliations:** 1 Labsystems Research Laboratories Helsinki Finland; 2 Hartman Institute Department of Virology University of Helsinki Helsinki Finland; 3 Meiji Institute of Health Science Odawara Japan; 4 Department of Virology Aurora Hospital Helsinki Finland; 5 Department of Chemistry University of Kuopio Box 1627 Kuopio 70211 Finland

## Abstract

*Objective:* We have evaluated the sensitivity and specificity of a new synthetic peptide-based species-specific enzyme immunoassay (EIA) for detection of *Chlamydia trachomatis* IgG and IgA antibodies.

*Methods:* Synthetic peptides derived from variable domain IV of major outer membrane protein (MOMP) were used as antigen in indirect EIA. IgG and IgA antibodies were measured in parallel with serum samples from *C. trachomatis* culture positive, culture negative, and antigen positive patients, and women with suspected *C. trachomatis* infection and blood donors. Sera from children under 15 years of age were used as controls.

*Results:* Culture positive women, culture positive men, and antigen positive women had positive peptide serology in 84.2%, 61.3%, and 93.1% of the cases, respectively. Among *C. trachomatis* suspected women, the antibody prevalence was 63.6%. Randomly collected blood donors showed a prevalence of 21.5%. Children with *C. pneumoniae* antibodies determined with the microimmuno-fluorescence (MIF) method did not show any reactivity in the *C. trachomatis* peptide EIA.

*Conclusions:* The results suggest that the new EIA test is highly specific for *C. trachomatis*, and *C. pneumoniae* antibodies do not interfere. Both IgG and IgA antibodies appear within at least 2 weeks in acute phase of infection among both culture positive and culture negative patients.


Infectious Diseases in Obstetrics and Gynecology 5:349–354 (1997)
© 1998 Wiley-Liss, Inc.

### Detection of Antibodies to *Chlamydia trachomatis* With Peptide-Based Species-Specific Enzyme Immunoassay

Ale Närvänen,[1]* Mirja Puolakkainen,[2] Wu Hao,[1] Kohsuke Kino,[3] and Jukka Suni[4]

[1]*Labsystems Research Laboratories, Helsinki, Finland*
[2]*Hartman Institute, Department of Virology, University of Helsinki, Helsinki, Finland*
[3]*Meiji Institute of Health Science, Odawara, Japan*
[4]*Department of Virology, Aurora Hospital, Helsinki, Finland*

### Abstract

***Objective:*** We have evaluated the sensitivity and specificity of a new synthetic peptide-based species-specific enzyme immunoassay (EIA) for detection of *Chlamydia trachomatis* IgG and IgA antibodies.

***Methods:*** Synthetic peptides derived from variable domain IV of major outer membrane protein (MOMP) were used as antigen in indirect EIA. IgG and IgA antibodies were measured in parallel with serum samples from *C. trachomatis* culture positive, culture negative, and antigen positive patients, and women with suspected *C. trachomatis* infection and blood donors. Sera from children under 15 years of age were used as controls.

***Results:*** Culture positive women, culture positive men, and antigen positive women had positive peptide serology in 84.2%, 61.3%, and 93.1% of the cases, respectively. Among *C. trachomatis* suspected women, the antibody prevalence was 63.6%. Randomly collected blood donors showed a prevalence of 21.5%. Children with *C. pneumoniae* antibodies determined with the microimmunofluorescence (MIF) method did not show any reactivity in the *C. trachomatis* peptide EIA.

***Conclusions:*** The results suggest that the new EIA test is highly specific for *C. trachomatis,* and *C. pneumoniae* antibodies do not interfere. Both IgG and IgA antibodies appear within at least 2 weeks in acute phase of infection among both culture positive and culture negative patients. Infect. Dis. Obstet. Gynecol. 5:349–354, 1997. © 1998 Wiley-Liss, Inc.

Key words
species specificity; enzyme immunoassay; serology; IgG; IgA

*Chlamydia trachomatis* is a leading cause of sexually transmitted disease, frequently occurring worldwide.[1] It has been estimated that 25% of infected men and 50–80% of women may be asymptomatic.[2–4] These individuals have a risk to further transmit the infection without any knowledge of the disease. In addition, the asymptomatic infection may lead to severe complications including infertility due to tubal damage, chronic abdominal pain, or chronic arthritis. Testing first void urine, made possible by the sensitive assays relying on the amplification of chlamydial DNA,[5,6] complements the earlier established methods, e.g., isolation in cell culture and detection of chlamydial antigen.[7,8] The diagnosis of past or complicated infections is not usually possible by using these methods as swabbing the infected site is not possible without invasive procedures or

*Correspondence to: Dr. Ale Närvänen, Department of Chemistry, University of Kuopio, Box 1627, 70211 Kuopio, Finland. E-mail: ale.narvanen@uku.fi

Received 26 February 1997
Accepted 25 September 1997

Clinical Study chlamydial particles may not be present at the site.

Another method to diagnose complicated chlamydial infections is to measure antibodies against *C. trachomatis* in serum or secretions.[9] The "gold standard" of chlamydia serology is the microimmunofluorescence (MIF) test.[10] Several enzyme immunoassay (EIA)-based tests have been developed for the detection of *C. trachomatis* antibodies.[11,12] However, during chlamydial infection, antibodies are also formed against the common antigenic epitopes present in all members of the genus *Chlamydia*. When whole bacteria are used as antigen in EIAs, the detection of these cross-reacting antibodies makes species-specific diagnosis difficult.

We have developed a synthetic peptide-based *C. trachomatis* species-specific EIA test to detect IgG and IgA antibody responses in humans. The peptides used are derived from the variable domain IV (VD IV) region of the major outer membrane protein (MOMP) of *C. trachomatis*. The synthesized peptides do not share any sequence homology with *C. pneumoniae* MOMP.

### SUBJECTS AND METHODS

### Peptide-Based EIAs

Species-specific *C. trachomatis* IgG and IgA EIAs are based on synthetic peptides derived from VD IV. Peptides were used as antigen in solid phase in conventional indirect EIA (Labsystems, Helsinki, Finland). Bound antibodies were detected either with anti-human-IgG-horseradish peroxidase (HRP) or with anti-human-IgA-HRP conjugates. Binding was visualized with tertramethyl benzidine (TMB) as a chromogen. Samples were diluted 10-fold (20 + 180 μl) in the sample diluent in both tests. The tests were run according to the manufacturer's instructions. The result was interpreted to be positive if the signal/cutoff value (S/C) was $\geq 1$ or negative if S/C was $<1$. *C. trachomatis* IgG or IgA positive human serum verified by MIF was used as the positive control in every run. Cutoff was defined 0.3 × absorbance value of the positive control.

### Patient Groups

Sera from patients visiting the Helsinki City Outpatient Clinic for Venereal Diseases with suspected *C. trachomatis* infection were studied. *C. trachomatis* culture results were available from these patients. Also paired sera collected at 2 week intervals were available from women and men with initially positive and negative *C. trachomatis* culture results. The first blood specimen was drawn at the same visit when the sample for *C. trachomatis* culture was taken. All cases were suspected to have an acute *C. trachomatis* infection. Sera from patients with positive chlamydial antigen detection test (Chugai/Gen-Probe, Tokyo, Japan) were from Motomura Obstetrics and Gynecology Clinic (Nagasaki, Japan), and samples from *C. trachomatis* suspected but not confirmed female age unknown were from Falco Commercial Laboratory (Kyoto, Japan).

As controls, sera from randomly collected (sex and age range unknown) blood donors from the Finnish Red Cross Blood Transfusion Center, children aged 1–7 years, collected during a hepatitis A virus (HAV) epidemic during autumn 1994 (children 1) from the Department of Virology, Aurora Hospital, and children aged 1–15 years with suspected viral and/or chlamydial illnesses (children 2) from the Department of Virology, University of Helsinki, were tested. Sera from the children 2 group were tested for the presence of *C. pneumoniae* antibodies by the MIF method developed at the Department of Virology, University of Helsinki. Antibodies of IgG and IgM classes to *C. pneumoniae* were detected in 46% and 14% of the cases, respectively.

### RESULTS

The *C. trachomatis* IgG and IgA antibody prevalence by the peptide-based EIA in different patient and control groups is presented in Table 1. 84.2% of *C. trachomatis* culture positive women, 61.3% of culture positive men, and 93.1% of *Chlamydia* antigen positive women were EIA positive. In the culture negative group, 45.3% of women and 38.3% of men were seropositive. A serostatus with an IgA antibody response but no IgG antibodies was more frequently seen in males than in females. Of culture negative men, 10.8% had detectable IgA antibodies without IgG response.

Randomly collected blood donors showed an antibody prevalence of 21.5%. Only two children had *C. trachomatis* antibodies detectable by the EIA. One was an IgG positive 6-month-old baby (children 1 group) with probable maternal antibodies to

**TABLE 1. Prevalence of *C. trachomatis* antibodies in different patient and control groups[a]**

| Group | N | Only IgG response (%) | Only IgA response (%) | IgG + IgA response (%) | Total response (%) |
|---|---|---|---|---|---|
| Women | | | | | |
| Culture positive | 82 | 13.4 | 2.4 | 68.3 | 84.2 |
| Antigen positive | 44 | 15.9 | 0.0 | 77.3 | 93.1 |
| Suspected only | 44 | 29.6 | 4.6 | 29.6 | 63.6 |
| Culture negative | 148 | 15.6 | 0.7 | 29.0 | 45.3 |
| Men | | | | | |
| Culture positive | 80 | 10.0 | 5.0 | 46.3 | 61.3 |
| Culture negative | 120 | 15.8 | 10.8 | 11.7 | 38.3 |
| Controls | | | | | |
| Blood donors | 247 | 11.3 | 5.3 | 4.7 | 21.5 |
| Children 1 | 67 | 1.5 | 0.0 | 0.0 | 1.5 |
| Children 2 | 85 | 0.0 | 1.2 | 0.0 | 1.2 |

[a]The total response is a sum of only IgG, only IgA, and simultaneous IgG and IgA (IgG + IgA) responses. Children 1 = children aged below 7 years, children 2 = children aged below 15 years, sera tested with *C. pneumoniae* MIF.

*C. trachomatis* and the other was an IgA antibody positive 15-year-old male (children 2 group) with a clinical diagnosis of reactive arthritis in the knee. This patient did not have *C. pneumoniae* IgG and IgM antibodies determined by MIF.

*C. trachomatis* antibody responses seen in paired sera from culture positive and culture negative patients of the sexually transmitted disease (STD) clinic are presented in Table 2. Patients were classified as follows: 1) stable IgG and IgA antibody titers (the change of S/C value was less than 2); 2) IgG and IgA antibody titer rises (the change of S/C value was more than 2); 3) seroconversions (the first sample was negative and second was positive); 4) negative (both samples were negative). All IgG titer rises were detected in the group of culture positive men. Seroconversion in IgG antibodies was found among culture positive patients; only one case was a women. Seroconversion in IgA antibodies was seen in three culture positive men and three culture negative women.

In the acute phase infection, IgG and IgA seroconversions or titer rises in the same patient were compared in Table 3. Two culture negative women with IgA seroconversion had already high IgG antibody levels in the first serum (F1 and F2). One culture negative women had an IgA seroconversion but remained IgG negative (F3). One culture positive women with IgG seroconversion was IgA negative (F4). All men with seroconversion were culture positive. Among the males with IgA seroconversion, two showed IgG titer rises (M1 and M2) and one IgG seroconversion (M3). The other male with IgG seroconversion had already stable IgA levels (M4). The two other IgG titer rises among the culture positive men showed either a decreasing (M5) or stable (M6) IgA level.

### DISCUSSION

Synthetic peptides offer an alternative method to create highly specific and sensitive serological tests for diagnosis of microbial infections.[13] They provide a possibility to design site-specific antigenic determinants for distinction between type-specific antibodies.[14] Since the MOMP of *C. trachomatis* is an antigenically complex protein having cross-reactive and species-specific epitopes,[15] the specificity can be achieved only by using synthetic peptide as an antigen.[16] For a more accurate determination of species-specific epitopes with the highest sensitivity, the continuous antigenic epitopes of *C. trachomatis* MOMP polypeptide were studied by using the epitope scanning method. Systematic scanning with overlapping peptides derived from the MOMP polypeptide showed species-specific antigenic determinants, which reacted with *C. trachomatis* antibody positive human sera.[17]

The difficulty in the serology of chlamydial infections is the close relationship of *C. trachomatis* and *C. pneumoniae.*[18] Common antigenic structures also in major polypeptides such as MOMP and kd60 may lead to the cross-reactions interfering with the diagnosis of *C. trachomatis* seroreactivity. Specificity of the assay is a question of importance in chlamydial serology since the epidemiology and transmission routes of *C. trachomatis* and *C. pneu-*

TABLE 2. Frequency of *C. trachomatis* antibody responses in paired sera from culture positive and culture negative STD patients in different phases

| | Culture positive | | | | Culture negative | | | |
|---|---|---|---|---|---|---|---|---|
| | Female | | Male | | Female | | Male | |
| Phase | IgG | IgA | IgG | IgA | IgG | IgA | IgG | IgA |
| Stable | 10 | 6 | 7 | 10 | 29 | 19 | 15 | 14 |
| 2 × titer rise | 0 | 0 | 4 | 0 | 0 | 0 | 0 | 0 |
| Seroconversion[a] | 1 | 0 | 2 | 3 | 0 | 3 | 0 | 0 |
| Negative | 1 | 5 | 2 | 2 | 30 | 36 | 18 | 19 |
| Total | 12 | 11 | 15 | 15 | 59 | 58 | 33 | 33 |

[a]The first sample is negative and the second sample is positive.

TABLE 3. Comparison of *C. trachomatis* IgG and IgA seroconversions or titer rise and culture result[a]

| | | S/C IgG | | S/C IgA | |
|---|---|---|---|---|---|
| Culture | Sex | 1st sample | 2nd sample | 1st sample | 2nd sample |
| Negative | F1 | 9.25 | 8.43 | 0.42 | 4.73 |
| | F2 | 7.06 | 6.73 | 0.31 | 1.90 |
| | F3 | 0.93 | 0.86 | 0.52 | 1.52 |
| Positive | F4 | 0.41 | 1.14 | 0.48 | 0.58 |
| | M1 | 1.36 | 3.11 | 0.66 | 1.12 |
| | M2 | 1.96 | 4.68 | 0.48 | 1.60 |
| | M3 | 0.76 | 8.97 | 0.27 | 1.54 |
| | M4 | 0.47 | 1.31 | 1.96 | 2.02 |
| | M5 | 1.27 | 2.89 | 4.19 | 2.03 |
| | M6 | 1.49 | 7.30 | 2.09 | 2.18 |

[a]F = female; M = male.

*moniae* infections are very different. The immunodominant epitopes derived from the VD IV region of *C. trachomatis* MOMP do not show any sequence homology with corresponding polypeptide of *C. pneumoniae*. The specificity of our assay was tested using serum samples from children aged 1–15 years. We selected these sera since most of the *C. trachomatis* infections are sexually acquired and are considered to be rare in this age group in Finland. The prevalence of *C. trachomatis*-specific antibodies among children was very low as expected: 2 of 152 children were seropositive. A 6-month-old baby had IgG antibodies, probably of maternal origin, and a 15-year-old male with reactive arthritis had IgA antibodies but not IgG antibodies. We could not ascertain whether this was a case of reactive arthritis following a genital infection. Although almost half of the children in group 2 had antibodies to *C. pneumoniae* by MIF, none of them had positive reaction in peptide EIA. This suggests that antibodies to *C. pneumoniae* do not interfere with *C. trachomatis* EIA.

To evaluate the specificity of the new assay, sera from unselected blood donors were tested by peptide EIA. The average age of Finnish blood donors is 40.3 years, with the frequency of men and women being 55% and 45%, respectively (Vähäsöyrinki, personal communication). The observed prevalence of *C. trachomatis* antibodies (IgG, IgA, or both) was 21.5% (Table 1). This is in agreement with an earlier study done in Finland: Saikku[19] has shown by using MIF that in patients with infection suspected to be of chlamydial origin the prevalence of both *C. trachomatis* and *C. pneumoniae* infections increases with the age. From age 30 years in both sexes the prevalence of *C. trachomatis* antibody is over 10%, reaching the maximum of almost 20% in women aged 31–40 years. Although men have more *C. pneumoniae* antibodies in all age groups, women have more *C. trachomatis* cases.[19]

The sensitivity of the peptide-based *C. trachomatis* EIA was tested with sera from Finnish female and male and Japanese female patients with an acute *C. trachomatis* genital infection. Among culture positive patients, the antibody prevalence was 84.2% in women and 61.3% in men. Of the 44 Japanese women with a positive cervical chlamydial antigen detection test, 93.2% had antibodies detectable by the peptide EIA. Patients with clinically suspected *C. trachomatis* infection that could not be confirmed by culture frequently had antibody response detectable by the EIA: 45.3% of the Finnish women, 63.6% of the Japanese women, and 38.3% of the Finnish men had *C. trachomatis* antibodies. This may reflect that while these patients did not have a confirmed infection during that visit, some of them might have had an infection earlier or they had had an acute infection that could not be detected due to insensitivity of the culture or the antigen detection tests.

There were several differences in the *C. trachomatis* antibody status between men and women. The seropositivity among the women with or without the culture confirmation was higher than that among men. This is in accordance with earlier reports on the antibody prevalence tested by MIF[10] suggesting that the antibodies are formed more often in women, perhaps due to the larger anatomical area affected. The difference also could be seen when testing paired sera. Most of the seroconversions and all of the titer rises occurred among culture positive men. All but one of the culture and antibody positive women had already developed IgG/IgA antibodies in the stable phase. This might suggest that either women on average are seroconverted earlier and faster or they become symptomatic later. Among culture negative patients there were three IgA seroconversions: two women had already stable IgG levels suggesting a reinfection and one was IgG negative, perhaps primary early stage infection.

Both culture positive and culture negative men more frequently showed a serostatus with IgA response without IgG antibodies than corresponding women (5.0% vs. 2.4% and 10.8% vs. 0.7%, Table 1). Among Japanese antigen positive women, no pure IgA response was found. In addition, three IgG seroconverted culture positive men had already stable or decreasing IgA levels.

There is evidence that serum IgA antibodies may be indicative of an active inflammatory process. IgA positivity has been associated with transference of the organism between partners as well as indirectly with women with evidence of tubal pathology.[20–24] Up to 80% of females with *C. trachomatis* infection have been reported to be asymptomatic.[2–4] This is supported by the finding that 10% of the blood donors in this study had IgA antibodies. This may indicate that the rate of chronic but asymptomatic infections is remarkable among the normal population. Since the consequences of chronic infection especially among women are severe, i.e., may lead to infertility, the screening of antibodies to *C. trachomatis* among the sexually active population is important.

### REFERENCES

1. Paavonen J, Wolner-Hanssen P: *Chlamydia trachomatis:* A major threat to reproduction. Hum Reprod 4:111–124, 1989.
2. Jaczek KH: Genital *Chlamydia trachomatis:* Detection, treatment, and patient education. Can Fam Phys 31: 1861–1865, 1985.
3. Stamm WE, Koutsky LA, Benedetti JK, Jourden JL, Brunham RC, Holmes KK: *Chlamydia trachomatis* urethral infections in men. Prevalence, risk factors, and clinical manifestations. Ann Intern Med 100:47–51, 1984.
4. Thejils H, Rahm VA, Rosen G, Gnarpe H: Correlation between *Chlamydia* infection and clinical evaluation, vaginal wet smear, and cervical swab test in female adolescents. Am J Obstet Gynecol 157:974–976, 1987.
5. Quinn TC, Welsh L, Lentz A, Crotchfelt K, Zenilman J, Newhall J, Gaydos C: Diagnosis by Amplicor PCR of *Chlamydia trachomatis* infection in urine samples from women and men attending sexually transmitted disease clinics. J Clin Microbiol 34:1401–1406, 1996.
6. Toye B, Peeling RW, Jessamine P, Claman P, Gemmil I: Diagnosis of *Chlamydia trachomatis* infections in asymptomatic men and women by PCR assay. J Clin Microbiol 34:1396–1400, 1996.
7. Pol van der B, Williams JA, Jones RB: Rapid antigen detection assay for identification of *Chlamydia trachomatis* infection. J Clin Microbiol 33:1920–1921, 1995.
8. Schachter J: Diagnosis of *Chlamydia trachomatis* infection. In Orfila J, Byrne GI, Chernesky MA, Grayston JT, Jones RB, Ridgway GL, Saikku P, Schachter J, Stamm WE, Stephens RS (eds): Chlamydial Infections: Proceedings of the 8th International Symposium on Human Chlamydial Infections. Bologna, Italy: Società Editrice Esculapio, pp 293–302, 1994.
9. Dabecause YAJM, Evers JLH, Land JA, Stals FS: *Chlamydia trachomatis* antibody testing is more accurate than hysterosalpingography in predicting tubal factor infertility. Fertil Steril 61:5:833–837, 1994.
10. Wang SP, Grayston JT: Micro-immunofluorescence serology of *Chlamydia trachomatis*. In de la Maza LM (ed): The 1983 International Symposium on Medical Virology. Amsterdam: Elsevier Biomedical Press, pp 87–118, 1984.
11. Clad A, Flecken U, Petersen EE: Chlamydial serology in genital infections: ImmunoComb versus Ipazyme. Infection 21:384–389, 1993.
12. Kumamoto Y, Sato T, Hiroi M, et al: Assessment of *Chlamydia trachomatis*-specific IgA and IgG serum antibodies in genitourinary *Chlamydia trachomatis* infection—Comparative study between HITAZYME and IPAzyme. Kansenshogaku Zasshi J Jpn Assoc Infect Dis 67:315–330, 1993.

13. Närvänen A, Korkolainen M, Suni J, et al.: Synthetic env gp41 peptide as a sensitive and specific diagnostic reagent in different stages of human immunodeficiency virus type 1 infection. J Med Virol 26:111–118, 1988.
14. Norrby E, Biberfeld G, Chiodi F, von Gegerfeldt A, Naucler A, Parks E, Lerner R: Discrimination between antibodies to HIV and to related retroviruses using site-directed serology. Nature 329:248–250, 1987.
15. Stephens RS, Tam MR, Kuo CC, Nowinski RC: Monoclonal antibodies to *Chlamydia trachomatis:* Antibody specificities and antigen characterization. J Immunol 128:1083–1089, 1992.
16. Jones HM, Schachter J, Stephen RS: Evaluation of the humoral immune response in trachoma in *Chlamydia trachomatis* major outer membrane protein by sequence-defined immunoassay. J Infect Dis 166:915–919, 1992.
17. Närvänen A, Seppänen H, Korkolainen M, et al.: Synthetic peptides in measuring human Chlamydia antibodies: Epitope mapping of major outer membrane protein of *Chlamydia trachomatis* serovar L2. In Mårdh PA, Saikku P (eds): Chlamydial Infections of the Genital and Respiratory Tracts and Allied Conditions. Uppsala, Sweden: Centre of STD Research, Uppsala University, pp 129–133, 1991.
18. Ward ME: The immunobiology and immunopathology of chlamydial infections. APMIS 103:769–796, 1995.
19. Saikku P: Diagnosis of acute and chronic *Chlamydia pneumoniae* infections. In Orfila J, Bryrne SI, Chernesky MA, et al. (ed): Chlamydial Infections: Proceedings of the 8th International Symposium on Human Chlamydial Infections. Bologna, Italy: Società Editrice Esculapio, pp 163–172, 1994.
20. Bjerke S, Purvis K: Characteristic of women under fertility investigation with IgA/IgG seropositivity for *Chlamydia trachomatis.* Eur J Obstet Gynecol Reprod Biol 51:157–161, 1993.
21. Cevenini R, Sarov I, Rumpianesi F, et al.: Serum specific IgA antibody to *Chlamydia trachomatis* in patients with chlamydial infections detected by ELISA and an immunofluorescence test. J Clin Pathol 37:686–691, 1984.
22. Eggert-Kruse W, Gerhard I, Naher H, Tigen W, Runnenbaum B: Chlamydial infection—A female and/or male infertility factor? Fertil Steril 53:1037–1043, 1990.
23. Nagy B, Gorradi G, Vadja Z, Gimes R, Csömör S: The occurrence of *Chlamydia trachomatis* in the semen of men participating in an IVF program. Hum Reprod 4:54–56, 1989.
24. Piura B, Sarov I, Sarov B, Kleinman D, Chaim W, Insler V: Serum IgG and IgA antibodies specific for *Chlamydia trachomatis* in salpingitis patients as determined by the immunoperoxidase assay. Eur J Epidemiol 1:110–116, 1985.

## References

[PDF_00468] Bjercke S., Purvis K. (1993). Characteristics of women under fertility investigation with IgA/IgG seropositivity for Chlamydia trachomatis.. Eur J Obstet Gynecol Reprod Biol.

[PDF_00472] Cevenini R., Sarov I., Rumpianesi F., Donati M., Melega C., Varotti C., La Placa M. (1984). Serum specific IgA antibody to Chlamydia trachomatis in patients with chlamydial infections detected by ELISA and an immunofluorescence test.. J Clin Pathol.

[PDF_00424] Clad A., Flecken U., Petersen E. E. (1993). Chlamydial serology in genital infections: ImmunoComb versus Ipazyme.. Infection.

[PDF_00416] Dabekausen Y. A., Evers J. L., Land J. A., Stals F. S. (1994). Chlamydia trachomatis antibody testing is more accurate than hysterosalpingography in predicting tubal factor infertility.. Fertil Steril.

[PDF_00477] Eggert-Kruse W., Gerhard I., Näher H., Tilgen W., Runnebaum B. (1990). Chlamydial infection--a female and/or male infertility factor?. Fertil Steril.

[PDF_00385] Jaczek K. H. (1985). Genital Chlamydia trachomatis: detection, treatment and patient education.. Can Fam Physician.

[PDF_00448] Jones H. M., Schachter J., Stephens R. S. (1992). Evaluation of the humoral immune response in trachoma to Chlamydia trachomatis major outer membrane proteins by sequence-defined immunoassay.. J Infect Dis.

[PDF_00428] Kumamoto Y., Sato T., Hiroi M., Hashizume S., Nakata H., Kojima H., Matsuda S., Yamazaki S., Sugao M., Noguchi M. (1993). Assessment of Chlamydia trachomatis-specific IgA and IgG serum antibodies in genitourinary Chlamydia trachomatis infection--comparative study between HITAZYME and IPAzyme.. Kansenshogaku Zasshi.

[PDF_00480] Nagy B., Corradi G., Vajda Z., Gimes R., Csömör S. (1989). The occurrence of Chlamydia trachomatis in the semen of men participating in an IVF programme.. Hum Reprod.

[PDF_00440] Norrby E., Biberfeld G., Chiodi F., von Gegerfeldt A., Nauclér A., Parks E., Lerner R. (1987). Discrimination between antibodies to HIV and to related retroviruses using site-directed serology.. Nature.

[PDF_00436] Närvänen A., Korkolainen M., Suni J., Korpela J., Kontio S., Partanen P., Vaheri A., Huhtala M. L. (1988). Synthetic env gp41 peptide as a sensitive and specific diagnostic reagent in different stages of human immunodeficiency virus type 1 infection.. J Med Virol.

[PDF_00382] Paavonen J., Wølner-Hanssen P. (1989). Chlamydia trachomatis: a major threat to reproduction.. Hum Reprod.

[PDF_00484] Piura B., Sarov I., Sarov B., Kleinman D., Chaim W., Insler V. (1985). Serum IgG and IgA antibodies specific for Chlamydia trachomatis in salpingitis patients as determined by the immunoperoxidase assay.. Eur J Epidemiol.

[PDF_00397] Quinn T. C., Welsh L., Lentz A., Crotchfelt K., Zenilman J., Newhall J., Gaydos C. (1996). Diagnosis by AMPLICOR PCR of Chlamydia trachomatis infection in urine samples from women and men attending sexually transmitted disease clinics.. J Clin Microbiol.

[PDF_00388] Stamm W. E., Koutsky L. A., Benedetti J. K., Jourden J. L., Brunham R. C., Holmes K. K. (1984). Chlamydia trachomatis urethral infections in men. Prevalence, risk factors, and clinical manifestations.. Ann Intern Med.

[PDF_00444] Stephens R. S., Tam M. R., Kuo C. C., Nowinski R. C. (1982). Monoclonal antibodies to Chlamydia trachomatis: antibody specificities and antigen characterization.. J Immunol.

[PDF_00393] Thejls H., Rahm V. A., Rosen G., Gnarpe H. (1987). Correlation between chlamydia infection and clinical evaluation, vaginal wet smear, and cervical swab test in female adolescents.. Am J Obstet Gynecol.

[PDF_00402] Toye B., Peeling R. W., Jessamine P., Claman P., Gemmill I. (1996). Diagnosis of Chlamydia trachomatis infections in asymptomatic men and women by PCR assay.. J Clin Microbiol.

[PDF_00406] Van der Pol B., Williams J. A., Jones R. B. (1995). Rapid antigen detection assay for identification of Chlamydia trachomatis infection.. J Clin Microbiol.

[PDF_00460] Ward M. E. (1995). The immunobiology and immunopathology of chlamydial infections.. APMIS.

